# Risk of embolic events before and after antibiotic treatment initiation among patients with left-side infective endocarditis

**DOI:** 10.1007/s15010-023-02066-z

**Published:** 2023-07-04

**Authors:** Matthaios Papadimitriou-Olivgeris, Benoit Guery, Nicoleta Ianculescu, Denise Auberson, Piergiorgio Tozzi, Matthias Kirsch, Pierre Monney

**Affiliations:** 1 Infectious Diseases Service, Lausanne University Hospital, University of Lausanne, 1011 Lausanne, Switzerland; 2 Department of Cardiology, Lausanne University Hospital, University of Lausanne, Lausanne, Switzerland; 3 Department of Cardiac Surgery, Lausanne University Hospital, University of Lausanne, Lausanne, Switzerland

**Keywords:** *Staphylococcus aureus*, Infective endocarditis, Embolization, Vegetation, Valvular surgery

## Abstract

**Purpose:**

Embolic events (EEs) are a common complication of left-side infective endocarditis (IE). The aim of the present study was to identify risk factors for the occurrence of EEs before or after antibiotic treatment instauration among patients with definite or possible IE.

**Methods:**

This retro-prospective study was conducted at the Lausanne University Hospital, Lausanne, Switzerland, from January 2014 to June 2022. EEs and IE were defined according to modified Duke criteria.

**Results:**

A total of 441 left-side IE episodes were included (334: 76% were definite and 107; 24% possible IE). EE were diagnosed in 260 (59%) episodes; in 190 (43%) before antibiotic treatment initiation and 148 (34%) after. Central nervous system (184; 42%) was the most common site of EE. Multivariable analysis identified *S. aureus* (*P* 0.022), immunological phenomena (*P* < 0.001), sepsis (*P* 0.027), vegetation size ≥ 10 mm (*P* 0.003) and intracardiac abscess (*P* 0.022) as predictors of EEs before antibiotic treatment initiation. For EEs after antibiotic treatment initiation, multivariable analysis revealed vegetation size ≥ 10 mm (*P* < 0.001), intracardiac abscess (*P* 0.035) and prior EE (*P* 0.042), as independent predictors of EEs, while valve surgery (*P* < 0.001) was associated with lower risk for EEs.

**Conclusions:**

We reported a high percentage of EEs among patients with left-side IE; vegetation size, intracardiac abscess, *S. aureus* and sepsis were independently associated with the occurrence of EEs. In addition to antibiotic treatment, early surgery led to further decrease in EEs incidence.

## Introduction

Although infective endocarditis (IE) is a rare disease with 1.5–11.6 cases per 100,000 people [1], it remains a life-threatening condition associated with an in-hospital mortality ranging from 15% to 30% and an 1-year mortality approaching 50% [2]. Embolic events (EEs) are one of the most common complications (21–50%) associated with IE and have a great influence on patients’ outcome [3–7]. A decrease in the EE’s incidence after antibiotic treatment initiation was previously described in several previous studies. However, most of these studies were performed more than two decades ago, when imaging for EEs detection was less frequently performed; therefore, asymptomatic EEs were less likely to be detected [3, 4, 8–10]. In addition, several studies also accepted diagnosis of EEs (such as stroke) only based on clinical symptoms/signs, without formal confirmation by imaging [4, 8, 10].

Many predictors of EEs have been previously identified, the most important among them being vegetation size > 10 mm [2, 4, 6, 7, 11, 12], *S. aureus* IE [6–8, 11], and prior EEs [4, 13, 14]. Early recognition of such predictors might help clinicians to identify patients, who could benefit from early surgery in order to prevent further embolization. To date, only the size of vegetation with or without severe valvular dysfunction and prior EEs are recognized surgical indications to prevent further EEs according to from the European Society of Cardiology (ESC) guidelines, since previous studies have shown a beneficial effect of early surgery on occurrence of EEs [15, 16].

The aim of the present study was to identify risk factors associated with the occurrence of EEs both before and after antibiotic treatment instauration.

## Materials and methods

### Study design

This study was conducted at the Lausanne University Hospital, Lausanne, Switzerland, a 1100-bed primary and tertiary care hospital from January 2014 to June 2022 (2014–17: retrospective cohort; 2018 onwards: prospective cohort).

### Patients

Inclusion criteria were adult patients (≥ 18 years old) and left-side IE according to modified Duke criteria. Additional inclusion criterion for the prospective cohort was the written consent and for the retrospective cohort the absence of refusal of the use of their data. A subsequent episode was excluded if it occurred within two months from the initial one.

Data regarding demographics (age, sex), comorbidities, cardiac predisposing factors [15], cardiac implantable electronic devices (CIEDs), microbiologic etiology, systemic symptoms, fever, acute heart failure, sepsis or septic shock, heart murmur, immunological phenomena [15], site of cardiac involvement and type of lesion (according to cardiac imaging modalities, macroscopic lesions on surgery or autopsy), cardiac surgery (timing), results of thoracoabdominal and cerebral imaging studies, embolic events (type, timing, symptoms) were retrieved from patients’ electronic health records. Study data were collected and managed using REDCap by an infectious diseases specialist. REDCap electronic data capture tools is hosted at Lausanne University Hospital. REDCap (Research Electronic Data Capture) is a secure, web-based software platform designed to support data capture for research studies [17, 18].

### Management of IE

According to internal guidelines, an infectious diseases consultation with a thorough physical examination was performed on a mandatory basis for all patients with IE suspicion. Thoraco-abdominal (computed tomography, abdominal magnetic resonance imaging or ^18^F-fluorodeoxyglucose positron emission positron emission tomography–computed tomography) and cerebral imaging (computed tomography or magnetic resonance imaging) were performed in all patients with clinical suspicion of EE based on local symptoms. Their realization in asymptomatic patients was left at the discretion of the treating physician and infectious diseases consultant. An endocarditis-team was established on January 2018, comprising of infectious diseases specialists, cardiologists, cardiac surgeons, which reviewed all patients with IE suspicion during weekly meetings.

### Definitions

The definition of EEs included major peripheral artery embolism, septic lung emboli, hepatic, renal or splenic emboli, mycotic aneurysm, ischemic or hemorrhagic stroke, cerebral abscess, conjunctival bleeding, retinal emboli, chorioretinitis, Janeway lesions or nail bed bleeding. EEs were divided according to their timing of occurrence into EE at those presented before versus after administration of antibiotic therapy. EEs were considered symptomatic if the patient presented local symptoms, such as confusion, headache, seizures, neurologic deficit for central nervous system EE, dyspnea, thoracic pain, cough for intrathoracic EE or abdominal pain, back pain for intraabdominal EE. Cutaneous EE (Janeway lesions or nail bed hemorrhages) were considered asymptomatic. For symptomatic EE, the date of EE was defined as the date of symptoms’ onset attributed to EE as reported by the patient.

IE was defined according to the ESC modified Duke criteria [15]. IE was characterized as community, healthcare or nosocomial according to Friedman et al. [19] Infection was categorized as sepsis or septic shock according to definition proposed by the Sepsis-3 International Consensus [20]. Valvular surgery within after antibiotic treatment initiation was included. A subset of patients that benefited from surgery but only if it was performed before the occurrence of EE after antibiotic treatment initiation was also included.

### Endpoint

The primary endpoint was incidence of EEs occurring within two months after the initiation of antibiotic therapy. Patients were followed until two months after antibiotic initiation (medical records review or telephone call) or death.

### Analysis

SPSS version 26.0 (SPSS, Chicago, IL, USA) software was used for data analysis. Categorical variables were analyzed using the *chi*-square or Fisher exact test and continuous variables with Mann–Whitney *U* test. Variables in bivariate analyses with *P* < 0.1 that did not contribute to multicollinearity were entered into the multivariable analyses. After checking Cox assumptions, two multivariable Cox proportional hazards (PH) regression models were performed with dependent variables being overall EEs and EEs occurring after antibiotic administration; for both models, valve surgery was treated as a time dependent covariable. Bivariate multivariable logistic regression analysis was performed with dependent variable being EEs occurring before antibiotic administration. Adjusted odds ratios (aORs) and 95% confidence intervals (CIs) were calculated to evaluate the strength of any association. All statistic tests were two-tailed and *P* < 0.05 was considered statistically significant. Kaplan–Meier curve of the embolic event probability after 4 days on antibiotic treatment of patients with left-side IE was performed for patients in order to assess the role of valvular surgery within 4 days after antibiotic treatment instauration.

## Results

### Study population

A total of 441 left-side IE episodes were included in 393 patients, among which, 334 (76%) were definite left-side IE and the remaining 107 (24%) possible. The prospective cohort included 274 (62%) episodes and the retrospective 167 (38%). In total, 182 (41%) patients developed sepsis, which was more common in patients with *S. aureus* IE (57% versus 24%; *P* < 0.001).

Valvular surgery within 2 months after antibiotic treatment initiation was performed in 189 (43%) episodes; 62 (14%) episodes underwent valvular surgery within 4 days after antibiotic treatment initiation. In total, 83 (19%) patients died within 2 months after antibiotic treatment initiation.

### Imaging studies

The assessment of cardiac involvement by IE was performed by transthoracic echocardiography (TTE) in 412 (93%) episodes, transesophageal echocardiography (TOE) in 356 (81%), ^18^F-Fluorodeoxyglucose Positron Emission Tomography/Computed Tomography (^18^F-FDG PET/CT) in 92 (21%), cardiac-CT in 29 (7%), macroscopic evaluation during surgery in 189 (43%) or autopsy in 9 (2%). Thoracoabdominal (CT scan or ^18^F-FDG PET/CT) or cerebral (CT scan or MRI) imaging studies were performed in 353 (80%) and 299 (68%) episodes, respectively.

### Embolic events

**Fig. 1:**
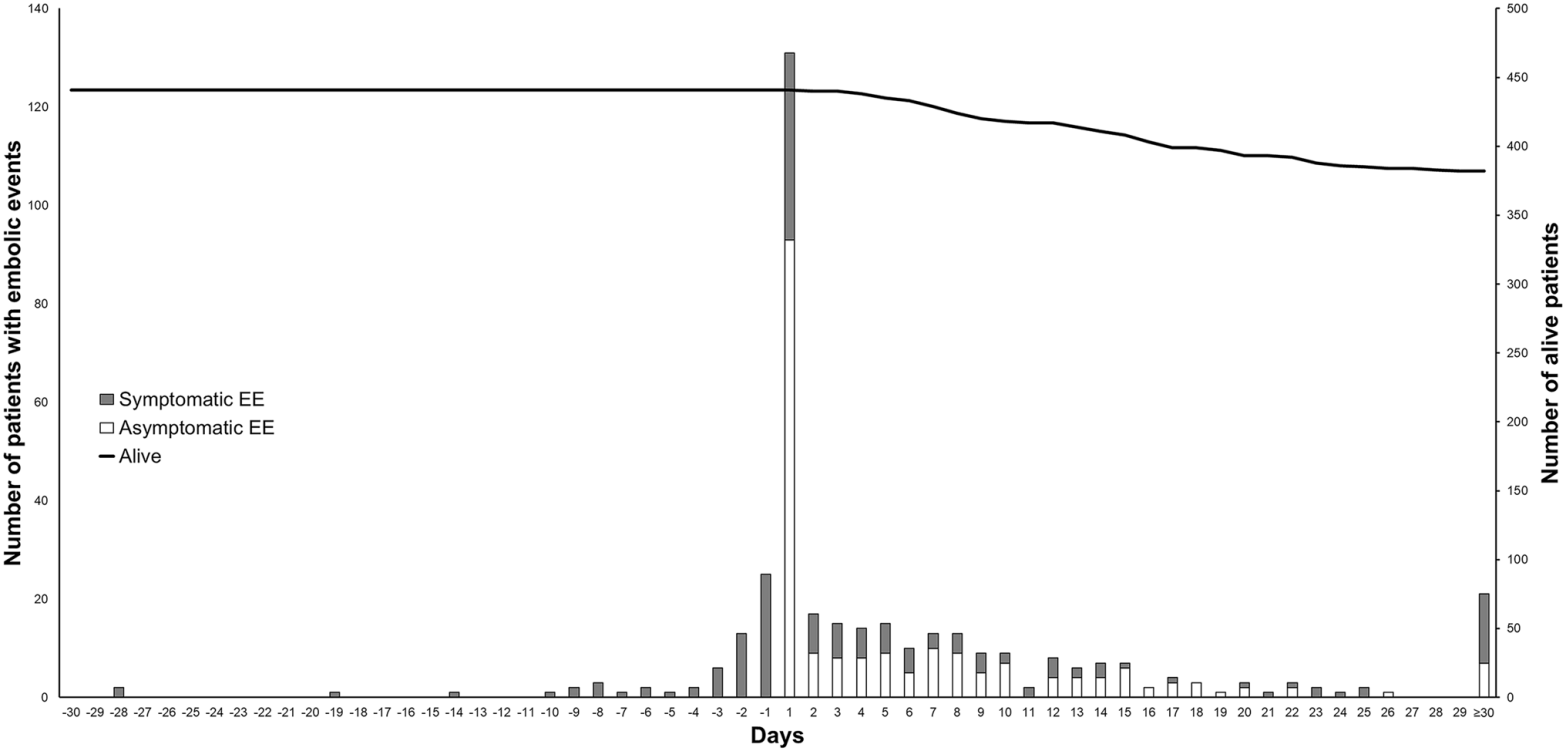
Timing of embolic events in relation to antibiotic treatment initiation

In total, EE were diagnosed in 260 (59%) episodes; they occurred in 190 (43%) before and in 148 (34%) after antibiotic treatment initiation (Fig. 1). EE were symptomatic in 98 (52%) before and in 66 (45%) after antibiotic treatment initiation. During the first week after antibiotic treatment, 97 (22%) patients developed a new EE; 48/424 (11%) developed an EE during the second week and 44/403 (11%) during the following 6 weeks.

### Site of embolic events

Central nervous system (184; 42%) was the most common site of EE, followed by the spleen (72; 16%). Table 1 shows the site of EEs before and after antibiotic treatment initiation. A thoracoabdominal imaging study was performed in 353 (80%) episodes (in the absence of symptoms in 196; 56%) and a cerebral imaging in 299 (68%) episodes (in the absence of symptoms in 138; 46%) (Table 2). EEs discovery by thoracoabdominal imaging studies did not differ among patients with and without symptoms (47% versus 45%; *P* 0.731). Cerebral imaging studies were more prone to detect EEs in symptomatic patients than in asymptomatic ones (68% versus 31%; *P* < 0.001).

**Table 1:** Site of embolic events before and after antibiotic treatment initiation in 441 patients with left-side infective endocarditis

	Before antibiotic treatment initiation	After antibiotic treatment initiation	Total
At least one embolic event	190 (43%)	148 (34%)	260 (59%)
Central nervous system	106 (24%)	108 (25%)	184 (42%)
Ischemic stroke	96 (22%)	90 (20%)	162 (37%)
Hemorrhagic stroke	18 (4%)	15 (3%)	33 (8%)
Cerebral abscess	0 (0%)	4 (1%)	4 (1%)
Cerebral mycotic aneurysm	8 (2%)	8 (2%)	15 (4%)
Ocular^a^	16 (4%)	5 (1%)	20 (5%)
Pulmonary septic emboli	17 (4%)	10 (2%)	26 (6%)
Intrabdominal organs			
Spleen	50 (11%)	25 (6%)	72 (16%)
Kidneys	33 (8%)	9 (2%)	41 (9%)
Liver	3 (1%)	4 (1%)	7 (2%)
Janeway lesions	51 (12%)	2 (1%)	51 (12%)
Subungual (splinter) hemorrhages	9 (2%)	0 (0%)	9 (2%)
Major artery occlusions	15 (3%)	10 (2%)	24 (5%)
Non-cerebral mycotic aneurysm	5 (1%)	10 (2%)	15 (3%)

^a^Conjunctival hemorrhages, retinal infractions, endophthalmitis

**Table 2:** Predictors of all embolic events (before and after antibiotic treatment initiation) and results of the Cox PH multivariable regression

	Without embolic event (*n* = 181)		Embolic event (*n* = 260)		*P*	Cox PH multivariable regression	
						*P*	aOR (95% CI)
Demographics							
Male sex	137	76%	200	77%	0.820		
Age (years)	70	57–80	67	54–75	0.004		
Age > 60 years	130	72%	169	65%	0.147		
Co-morbidities							
Atrial fibrillation	53	29%	62	24%	0.226		
Congestive heart failure	17	9%	22	8%	0.736		
Cirrhosis	9	5%	20	8%	0.330		
Diabetes mellitus	37	20%	74	28%	0.059	0.384	1.13 (0.86–1.50)
Chronic kidney disease (moderate or severe)	39	22%	41	16%	0.133		
Immunosuppression	20	11%	20	8%	0.241		
Setting of infection onset							
Community or non-nosocomial healthcare-associated	153	85%	233	90%			
Nosocomial	28	15%	27	10%	0.142		
Cardiac predisposing factors	93	51%	129	50%	0.772		
IV drug use	4	2%	21	8%	0.011	0.091	1.50 (0.94–2.39)
Prior endocarditis	17	9%	25	10%	1.000		
Native valve disease	26	14%	34	13%	0.778		
Prosthetic valve	60	33%	74	28%	0.295		
Cardiac implantable electronic devices	24	13%	30	12%	0.658		
Timing of IE							
2014–2017 (retrospective cohort)	64	35%	103	40%			
2018–2022 (prospective cohort)	117	65%	157	60%	0.371		
Microbiological data							
*S. aureus*	59	33%	105	40%	0.109		
Coagulase negative staphylococci	15	8%	10	4%	0.059		
Streptococci	50	28%	80	31%	0.525		
Enterococci	27	15%	35	13%	0.678		
Other Gram-positive	10	6%	5	2%	0.059		
HACEK	8	4%	8	3%	0.453		
Other Gram-negative	7	4%	4	2%	0.134		
Intracellular pathogens	1	1%	4	2%	0.653		
Fungi	2	1%	2	1%	1.000		
Polymicrobial infection	9	5%	3	1%	0.019		
No identification	11	6%	10	4%	0.364		
Manifestations							
Systemic symptoms	169	93%	243	93%	1.000		
Fever	148	82%	213	82%	1.000		
Heart murmur	114	63%	163	63%	1.000		
New heart murmur	76	42%	122	47%	0.331		
Acute heart failure prior to antibiotic treatment	41	23%	56	22%	0.816		
Immunologic phenomena	5	3%	34	13%	< 0.001	0.002	1.81 (1.25–2.61)
Sepsis	60	33%	122	47%	0.004	0.026	1.33 (1.04–1.72)
Septic shock	18	10%	49	19%	0.011		
New second or third degree atrioventricular bloc	5	3%	7	3%	1.000		
Site of infection							
Aortic valve	114	63%	146	56%	0.169		
Mitral valve	78	43%	138	53%	0.042	0.491	1.10 (0.84–1.43)
Other left-side site of infection	3	2%	1	0%	0.310		
Right-side valve	1	1%	13	5%	0.010		
Multivalvular	15	8%	38	15%	0.053	0.397	1.18 (0.81–1.71)
CIED-IE	2	1%	9	3%	0.213		
Type of left-side valve							
Native	127	70%	195	75%	0.277		
Prosthetic	52	29%	68	26%	0.587		
Positive imaging and/or pathological modified Duke criteria	118	65%	205	79%	0.002		
Type of left-side intracardiac lesions							
Vegetation	93	51%	190	73%	< 0.001		
Vegetation size (mm)	7	3–12	12	6–17	< 0.001		
Vegetation ≥ 10 mm	33	18%	121	47%	< 0.001	< 0.001	1.68 (1.29–2.18)
Abscess	26	14%	73	28%	0.001	0.019	1.42 (1.06–1.90)
Perforation	16	9%	37	14%	0.102		
Dehiscence of prosthetic valve	6	3%	10	4%	1.000		
Fistula	3	2%	6	2%	0.743		
Pseudoaneurysm	5	3%	6	2%	0.766		
Aneurysm	0	0%	1	0%	1.000		
Severe valvular regurgitation	63	35%	105	40%	0.273		
Valve surgery	56	31%	133	51%	< 0.001		
Valve surgery in patients without embolic event before antibiotic treatment initiation	56	31%	30	12%	< 0.001	0.038	0.37 (0.15–0.95)^a^

Data are depicted as number/percentage or median/Q1–Q3

*CIED* cardiac implantable electronic devices; *CRP* C-reactive protein; *HACEK Haemophilus* spp., *Aggregatibacter* spp., *Cardiobacterium hominis*, *Eikenella corrodens*, *Kingella kingae*; *IE* infective endocarditis; *PH* proportional hazard

^a^Treated as time dependent covariable

### Predictors of embolic events

Table 2 summarizes the factors associated with overall EE (before and after antibiotic treatment initiation). Cox PH regression model (Table 2) identified immunologic phenomena (*P* 0.002; aOR 1.81, CI 1.25–2.61), sepsis (*P* 0.026; aOR 1.33, CI 1.04–1.72), vegetation size ≥ 10 mm (*P* < 0.001; aOR 1.68, CI 1.29–2.18) and intracardiac abscess (*P* 0.019; aOR 1.42, CI 1.06–1.90) as independent predictors of EEs in patients with left-side IE, while valve surgery (*P* 0.019; OR 1.42, CI 1.06–1.90) was associated with lower risk of EEs.

Table 3 summarizes the factors associated with EE identified before antibiotic treatment initiation. *S. aureus* (*P* 0.022; aOR 1.76, CI 1.09–2.86), immunological phenomena (*P* < 0.001; aOR 6.99, CI 2.91–16.80), sepsis (*P* 0.027; aOR 1.76, CI 1.09–2.66), vegetation size ≥ 10 mm (*P* 0.003; aOR 2.02, CI 1.26–3.22) and intracardiac abscess (*P* 0.022; aOR 1.87, CI 1.09–3.21) were associated with EEs before antibiotic treatment initiation (Table 3).

**Table 3:** Predictors of embolic events before antibiotic treatment initiation and results of the multivariable logistic regression

	Without embolic event (*n* = 251)		Embolic event (*n* = 190)		*P*	Multivariable logistic regression	
						*P*	aOR (95% CI)
Demographics							
Male sex	191	76%	146	77%	0.910		
Age (years)	70	58–79	65	51–74	< 0.001		
Age > 60 years	185	74%	114	60%	0.003	0.072	0.63 (0.38–1.04)
Co-morbidities							
Atrial fibrillation	78	31%	37	19%	0.006	0.307	0.76 (0.45–1.28)
Congestive heart failure	27	11%	12	6%	0.128		
Cirrhosis	16	6%	13	7%	0.849		
Diabetes mellitus	65	26%	46	24%	0.740		
Chronic kidney disease (moderate or severe)	51	20%	29	15%	0.212		
Immunosuppression	30	12%	10	5%	0.018	0.099	0.51 (0.23–1.14)
Setting of infection onset							
Community or non-nosocomial healthcare-associated	211	84%	175	92%			
Nosocomial	40	16%	15	8%	0.013	0.075	0.52 (0.26–1.07)
Cardiac predisposing factors	128	51%	94	49%	0.774		
IV drug use	9	4%	16	8%	0.037	0.616	1.29 (0.48–3.46)
Prior endocarditis	27	11%	15	8%	0.331		
Native valve disease	32	13%	28	15%	0.576		
Prosthetic valve	84	33%	50	26%	0.117		
Cardiac implantable electronic devices	38	15%	16	8%	0.040		
Timing of IE							
2014–2017 (retrospective cohort)	92	37	75	40			
2018–2022 (prospective cohort)	159	63	115	61	0.306		
Microbiological data							
*S. aureus*	82	33%	82	43%	0.029	0.022	1.76 (1.09–2.86)
Coagulase negative staphylococci	18	7%	7	4%	0.146		
Streptococci	70	28%	60	32%	0.402		
Enterococci	38	15%	24	13%	0.491		
Other Gram-positive	10	4%	5	3%	0.598		
HACEK	10	4%	6	3%	0.799		
Other Gram-negative	10	4%	1	1%	0.028		
Intracellular pathogens	2	1%	3	2%	0.656		
Fungi	3	1%	1	1%	0.638		
Polymicrobial infection	9	4%	3	2%	0.247		
No identification	17	7%	4	2%	0.024		
Manifestations							
Systemic symptoms	231	92%	181	95%	0.244		
Fever	205	82%	156	82%	1.000		
Heart murmur	154	61%	123	65%	0.487		
New heart murmur	104	41%	94	49%	0.101		
Acute heart failure prior to antibiotic treatment	58	23%	39	21%	0.562		
Immunologic phenomena	8	3%	31	16%	< 0.001	< 0.001	6.99 (2.91–16.80)
Sepsis	91	36%	91	48%	0.015	0.027	1.76 (1.09–2.66)
Septic shock	33	13%	34	18%	0.182		
New second or third degree atrioventricular bloc	8	3%	4	2%	0.566		
Positive imaging and/or pathological modified Duke criteria	170	68%	153	82%	0.003		
Site of infection							
Aortic valve	156	62%	104	55%	0.119		
Mitral valve	111	44%	105	55%	0.027	0.063	1.54 (0.98–2.43)
Other left-side site of infection	3	1%	1	1%	0.638		
Right-side valve	3	1%	11	6%	0.011		
Multivalvular	22	9%	31	16%	0.018	0.512	1.25 (0.64–2.46)
CIED-IE	5	2%	6	3%	0.542		
Type of left-side valve							
Native	178	71%	144	76%	0.279		
Prosthetic	73	29%	47	25%	0.332		
Type of left-side intracardiac lesions							
Vegetation	141	56%	142	75%	< 0.001		
Vegetation size (mm)	8	4–14	11	7–17	0.016		
Vegetation ≥ 10 mm	64	25%	90	47%	< 0.001	0.003	2.02 (1.26–3.22)
Abscess	41	16%	58	31%	0.001	0.022	1.87 (1.09–3.21)
Perforation	26	10%	27	14%	0.238		
Dehiscence of prosthetic valve	9	4%	7	4%	1.000		
Fistula	4	2%	5	3%	0.508		
Pseudoaneurysm	8	3%	3	2%	0.364		
Aneurysm	0	0%	1	1%	0.431		
Severe valvular regurgitation	85	34%	83	44%	0.038	0.688	1.10 (0.68.–1.79)

Data are depicted as number/percentage or median/Q1–Q3

*CIED* cardiac implantable electronic devices; *CRP* C-reactive protein; *HACEK Haemophilus* spp., *Aggregatibacter* spp., *Cardiobacterium hominis*, *Eikenella corrodens*, *Kingella kingae*; *IE* infective endocarditis

**Fig. 2:**
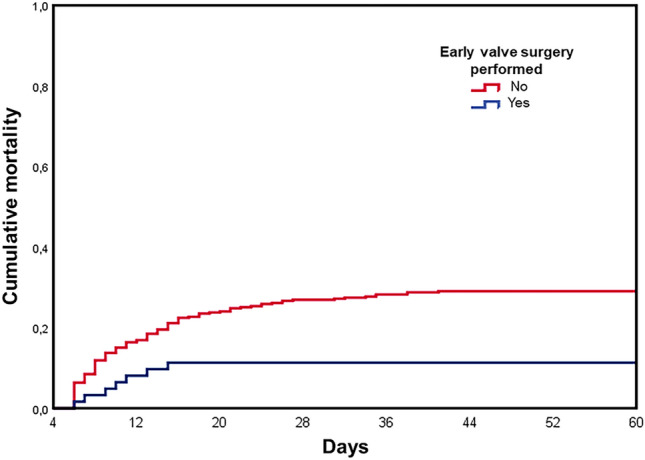
Kaplan–Meier curve of the embolic event probability after 4 days on antibiotic treatment of patients with left-side IE according to early (within 4 days after antibiotic treatment initiation) valve surgery

Table 4 summarizes the factors associated with new EE identified after antibiotic treatment initiation. Cox PH regression model (Table 4) revealed EE before antibiotic treatment initiation (*P* 0.042; OR 1.43, CI 1.13–2.02), sepsis (*P* 0.009; OR 1.56, CI 1.12–2.16), vegetation size ≥ 10 mm (*P* 0.005; OR 1.67, CI 1.17–2.39) and intracardiac abscess (*P* 0.035; OR 1.52, CI 1.03–2.25) as independent predictors of EEs after antibiotic treatment initiation, while valve surgery (*P* < 0.001; OR 0.40, CI 0.24–0.67) was associated with lower risk of EEs after antibiotic treatment initiation. Figure 2 shows a Kaplan–Meier curve of the embolic event probability after 4 days on antibiotic treatment of patients with left-side IE according to valvular surgery performed within 4 days after antibiotic treatment initiation; valvular surgery was associated with lower risk of embolic events (*P* 0.005).

**Table 4:** Predictors of embolic events after antibiotic treatment initiation and results of the Cox PH multivariable regression

	Without embolic events (*n* = 293)		Embolic events (*n* = 148)		*P*	Cox PH multivariable regression	
						*P*	aOR (95% CI)
Demographics							
Male sex	223	76%	114	77%	0.906		
Age (years)	68	55–77	68	56–76	0.484		
Age > 60 years	198	68%	101	68%	0.914		
Co-morbidities							
Atrial fibrillation	75	26%	40	27%	0.818		
Congestive heart failure	25	9%	14	9%	0.726		
Cirrhosis	13	4%	16	11%	0.014	0.098	1.58 (0.92–2.71)
Diabetes mellitus	68	23%	43	29%	0.202		
Chronic kidney disease (moderate or severe)	57	19%	23	16%	0.360		
Immunosuppression	26	9%	14	9%	0.861		
Setting of infection onset							
Community or non-nosocomial healthcare-associated	255	87%	131	89%			
Nosocomial	38	13%	17	11%	0.761		
Cardiac predisposing factors	148	51%	74	50%	0.920		
IV drug use	12	4%	13	9%	0.051	0.208	1.47 (0.81–2.69)
Prior endocarditis	27	9%	15	10%	0.735		
Native valve disease	43	15%	17	11%	0.382		
Prosthetic valve	88	30%	46	31%	0.827		
Cardiac implantable electronic devices	34	12%	20	14%	0.645		
Timing of IE							
2014–2017 (retrospective cohort)	108	37%	59	40%			
2018–2022 (prospective cohort)	185	63%	89	60%	0.603		
Microbiological data							
*S. aureus*	99	34%	65	44%	0.047		
Coagulase negative staphylococci	20	7%	5	3%	0.190		
Streptococci	87	30%	43	29%	0.912		
Enterococci	47	16%	15	10%	0.110		
Other Gram-positive	14	5%	1	1%	0.025		
HACEK	13	4%	3	2%	0.283		
Other Gram-negative	8	3%	3	2%	0.758		
Intracellular pathogens	2	1%	3	2%	0.340		
Fungi	2	1%	2	1%	0.605		
Polymicrobial infection	12	4%	0	0%	0.010		
No identification	13	4%	8	5%	0.642		
Manifestations							
Systemic symptoms	276	94%	136	92%	0.416		
Fever	241	82%	120	81%	0.794		
Heart murmur	191	65%	86	58%	0.175		
New heart murmur	135	46%	63	43%	0.543		
Acute heart failure prior to antibiotic treatment	65	22%	32	22%	1.000		
EE before antibiotic treatment initiation	112	38%	78	53%	0.004	0.042	1.43 (1.13–2.02)
Immunologic phenomena	23	8%	16	11%	0.374		
Sepsis	107	37%	75	51%	0.006	0.008	1.56 (1.12–2.16)
Septic shock	37	13%	30	20%	0.048		
New second or third degree atrioventricular bloc	6	2%	6	4%	0.229		
Positive imaging and/or pathological modified Duke criteria	212	72%	111	75%	0.571		
Site of infection							
Aortic valve	178	61%	82	55%	0.306		
Mitral valve	137	47%	79	53%	0.192		
Other left-side site of infection	3	1%	1	1%	1.000		
Right-side valve	8	3%	6	4%	0.566		
Multivalvular	33	11%	20	14%	0.536		
CIED-IE	6	2%	5	3%	0.519		
Type of left-side valve							
Native	214	73%	108	73%	1.000		
Prosthetic	78	27%	42	28%	0.734		
Type of left-side intracardiac lesions							
Vegetation	181	62%	102	69%	0.143		
Vegetation size (mm)	9	5–15	12	6–17	0.084		
Vegetation ≥ 10 mm	88	30%	66	45%	0.003	0.005	1.67 (1.17–2.39)
Abscess	57	19%	42	28%	0.040	0.035	1.52 (1.03–2.25)
Perforation	34	12%	19	13%	0.757		
Dehiscence of prosthetic valve	9	3%	7	5%	0.422		
Fistula	6	2%	3	2%	1.000		
Pseudoaneurysm	8	3%	3	2%	0.758		
Aneurysm	0	0%	1	1%	0.336		
Severe valvular regurgitation	116	40%	52	35%	0.406		
Valve surgery	118	40%	71	48%	0.128	< 0.001	0.40 (0.24–0.67)^a^
Valve surgery within 4 days	49	17%	13	9%	0.029		

Data are depicted as number/percentage or median/Q1–Q3

*CIED* cardiac implantable electronic devices; *CRP* C-reactive protein; *HACEK Haemophilus* spp., *Aggregatibacter* spp., *Cardiobacterium hominis*, *Eikenella corrodens*, *Kingella kingae*; *IE* infective endocarditis; *PH* proportional hazard

^a^Treated time dependent covariable

## Discussion

In the present study, at least one EE occurred in 59% of IE episodes, which was higher than previously reported (21–50%) [3–7]. In a meta-analysis, EEs median incidence was 29% [11]. A high percentage of patients (32%) presented symptomatic EE, as previously reported (21–37%) [3, 4, 12, 21, 22].

Regarding significant EE occurring after antibiotic administration, 20% developed at least one, which was higher than previously reported (7–14%) [3, 4, 6, 7, 12, 13, 21]. The incidence decreased progressively over time after the initiation of antibiotic treatment. Our results are in accordance to previous studies indicating that embolic risk decreases to half after one week of antimicrobial treatment [8, 10]. In the present study, in contrast to previous studies, the risk of EE remained present even after two weeks of treatment [10].

As previously reported, EE involved more commonly the central nervous system [3, 5–7, 22, 23]. The incidence of symptomatic central nervous system EEs (24%), were higher than previously reported (10–15%) [3–6, 8, 22]. A possible explanation could be that in our center a cerebral imaging study was systematically performed for any central nervous system symptom such as neurologic deficit, seizures, confusion or headache, while in previous studies, imaging studies were performed only in patients with a neurologic deficit [8]. After the antibiotic treatment, new symptomatic central nervous system EE occurred in 16%, which was also higher than previously reported (4–6%) [7, 8].

Previous studies showed an association between *S. aureus*, the most common cause of IE, and the risk of EEs [6–8, 11, 24]. In our study, *S. aureus* was associated with EE before antibiotic treatment initiation, but it did not influence the EEs’ risk after antibiotic initiation; this discordance was previously found in other studies [7].

Likewise, many studies have shown that vegetation’s size was a major determinant of EE risk [2, 4, 6, 7, 11, 12]. In the present study, vegetation size ≥ 10 mm was associated with the occurrence of EE both before and after antibiotic treatment initiation. We also found that the presence of intracardiac abscess was also associated with EE occurrence before or after antibiotic treatment initiation, a finding which was not observed in the meta-analysis, where intracardiac lesions other than vegetation had no significant influence on EE risk [11]. Intracardiac abscess was found to be associated with central nervous system EE after the initiation of antibiotic treatment in a study from the International Collaboration on Endocarditis Prospective Cohort Study [8]. Finally, even though mitral valve endocarditis was previously shown to be associated with high risk for EE [8, 11], this variable failed to achieve statistical significance in our multivariable model.

EEs at presentation were found to predispose to EEs after the initiation of antibiotic treatment, as previously reported [4, 13, 14]. Their importance was underlined by the fact that EEs at initial presentation are part of the risk prediction score for EEs after antibiotic treatment initiation [12]. Prior EEs in association with vegetation size > 10 mm is a recognized indication for valve surgery according to the ESC guidelines for the prevention of further embolism [15]. In the present study, a reduction in EEs was achieved with early surgery. While the performance of early surgery in patients with an operative indication was found in a meta-analysis to be associated with lower in-hospital and 1-year mortality as compared to patients treated with antimicrobial treatment only or antimicrobial treatment and late operation, the role of early surgery for the reduction of EE risk remains unclear [25]. The present study reinforces the role of early surgical management in patients with operative indication, in order to reduce the risk of further EEs, since 42% of patients with a vegetation > 10 mm developed an embolic event after antibiotic treatment initiation [16, 26, 27]. In the first randomized clinical trial of patients with large vegetations without heart failure, but at high-risk for EEs, early surgery resulted in significantly lower rate of EEs, as compared to conventional treatment (0% versus 21%; *P* 0.005) [16]. In the 2015 ESC guidelines, valve surgery is recommended for patients with a left-sided valve vegetation > 10 mm if an embolic event occurs after 5 days of appropriate antibiotic treatment [15]. Our data indicate that vegetations > 10 mm and EEs before antibiotic treatment initiation are both independently associated with EEs after antibiotic treatment initiation, and that valve surgery provides a significant reduction in the risk of subsequent EEs. Those observations suggest that the aforementioned surgical indication should be extended to patients with a vegetation > 10 mm and one or more embolic events, independently of the timing of the embolic event. Since the risk of EE during antibiotic treatment is higher in the two first weeks, surgery in patients with a surgical indication for embolism prevention, could be more beneficial if performed without delay. A clinical trial (Antibiotics vs Antibiotics and Surgical ThERapy for Infective Endocarditis: ASTERIx) is ongoing to determine the benefit of surgery in IE patients with a vegetation > 10 mm and one or no embolic events.

In the present study, sepsis was associated with EEs before antibiotic treatment initiation. To the best of our knowledge, this is the first study to show such an association. A possible explanation could be that EE and sepsis are part of more severe IE, caused by *S. aureus*. Indeed, in the present study sepsis was more common in patients with *S. aureus* IE, also being a predictor of EE. It was previously shown that patients with sepsis (qSOFA score ≥ 2) were at higher risk for adverse events, including EE [28].

The study has several limitations. First, it was monocentric, with almost one third of patients being retrospectively collected. Another limitation was that vegetation motility, that was previously found to be associated with EEs, was not evaluated in the present study [11]. Moreover, 19% of patients did not have a TOE, thus the calculation of the vegetation size was only based only on the TTE in those patients. A referral bias applied to the present study, since our center was the referral center for cardiac surgery. In addition, not all patients benefited from cerebral or cerebral imaging studies, thus the incidence of asymptomatic EE may have been underestimated [23]. Furthermore, we cannot exclude that the etiology of some EEs was other than IE (p.ex. atrial fibrillation).

In conclusion, we reported a high percentage of EEs (59%) among patients with left-side IE. Vegetation size, intracardiac abscess, IE due to *S. aureus* and sepsis were associated with occurrence of EEs. Even though EEs risk declined steadily during treatment, EEs remained a frequent occurrence, especially in patients with prior EEs. In addition to antibiotic treatment, early surgery, led to further decrease in EEs incidence.

## Data Availability

The data that support the findings of this study are available from the corresponding author upon reasonable request.
